# The Cancer Spliceome: Reprograming of Alternative Splicing in Cancer

**DOI:** 10.3389/fmolb.2018.00080

**Published:** 2018-09-07

**Authors:** Ettaib El Marabti, Ihab Younis

**Affiliations:** Biological Sciences Program, Carnegie Mellon University in Qatar, Doha, Qatar

**Keywords:** splicing, cancer spliceome, alternative splicing, exons, introns

## Abstract

Alternative splicing allows for the expression of multiple RNA and protein isoforms from one gene, making it a major contributor to transcriptome and proteome diversification in eukaryotes. Advances in next generation sequencing technologies and genome-wide analyses have recently underscored the fact that the vast majority of multi-exon genes under normal physiology engage in alternative splicing in tissue-specific and developmental-specific manner. On the other hand, cancer cells exhibit remarkable transcriptome alterations partly by adopting cancer-specific splicing isoforms. These isoforms and their encoded proteins are not insignificant byproducts of the abnormal physiology of cancer cells, but either drivers of cancer progression or small but significant contributors to specific cancer hallmarks. Thus, it is paramount that the pathways that regulate alternative splicing in cancer, including the splicing factors that bind to pre-mRNAs and modulate spliceosome recruitment. In this review, we present a few distinct cases of alternative splicing in cancer, with an emphasis on their regulation as well as their contribution to cancer cell phenotype. Several categories of splicing aberrations are highlighted, including alterations in cancer-related genes that directly affect their pre-mRNA splicing, mutations in genes encoding splicing factors or core spliceosomal subunits, and the seemingly mutation-free disruptions in the balance of the expression of RNA-binding proteins, including components of both the major (U2-dependent) and minor (U12-dependent) spliceosomes. Given that the latter two classes cause global alterations in splicing that affect a wide range of genes, it remains a challenge to identify the ones that contribute to cancer progression. These challenges necessitate a systematic approach to decipher these aberrations and their impact on cancer. Ultimately, a sufficient understanding of splicing deregulation in cancer is predicted to pave the way for novel and innovative RNA-based therapies.

## Splicing is an evolutionary conserved and essential step in gene expression in eukaryotes

Most genes in eukaryotes contain intervening sequences (introns) that disrupt the expressed sequences (exons). Introns in eukaryotes are much longer (median size ~1,000 bp but can be >100,000 bp) compared to exons (median size ~120 bp), making introns the major contributors to the sequence of genes. After transcription, in order for the expressed transcript (pre-mRNA) to become a suitable message for downstream processes such as translation of the encoded protein, the pre-mRNA from any multi-exon gene has to undergo extensive processing to remove the introns by an extraordinary molecular machine, the spliceosome. Given their large size, introns add a long time, sometimes hours, to the transcription process of genes in eukaryotes. Thus, introns present a conundrum as their transcription, just to be spliced out and degraded, seems to be a wasteful process both in terms of the time it takes to transcribe them and the energy consumed in their transcription as well as their removal and degradation. In addition, the splicing process needs to be extremely efficient as well as be executed with high fidelity. Efficiency is required to make sure all introns are removed from the pre-mRNA on time and in a coordinated manner. Fidelity is of paramount importance because joining exons with any mistake of even one base could have catastrophic effects on the reading frame. Furthermore, the cis-sequences or splice sites at the boundaries of each intron are too simple, sometimes degenerate and highly redundant outside the actual splice sites to serve alone as efficient landmarks for spliceosome assembly. Taken together, these features of introns and splicing in general make the presence of introns in eukaryotes counterintuitive. However, introns are not simply extra sequences that are removed by splicing, but rather have several advantages such as coupling multiple RNA processing events for higher gene expression efficiency as well as regulation and providing a checkpoint for quality control of the mRNA. They also allow any gene that harbor them to have a tremendous capacity for diversification through the process of alternative splicing. Thus, it is likely that the advantages of harboring introns outweigh the disadvantages as their presence in eukaryotic genomes and to some extent their position in the genes are highly conserved (Fedorov et al., 2002; Rogozin et al., 2003), in some cases between humans and the plant *Arabidopsis thaliana*.

## Diversification of transcriptomes by alternative splicing

The advent of high-throughput sequencing has uncovered the fact that most multi-exon genes in eukaryotes undergo at least one event of alternative splicing (Pan et al., 2008), generating two or more distinct mRNAs from the same gene, with the number of alternatively spliced transcripts potentially staggering for some genes. Interestingly, many such transcripts are expressed in a tissue-specific manner, at specific developmental stage, or in a disease-specific manner (Castle et al., 2008; Wang et al., 2008). While the function of some of these alternative transcripts is not always immediately interpretable or even recognized, a plethora of work indicates that alternatively spliced exons are translated and tend to encode important domains in the encoded polypeptide (Kalsotra and Cooper, 2011; Ellis et al., 2012; Weatheritt et al., 2016; Tapial et al., 2017). This suggests an evolutionary conserved molecular design for transcriptome diversification without the need to expand the genome that would require creating genes that are homologous to existing ones that serve similar yet distinct functions (Nilsen and Graveley, 2010).

**Figure 1:**
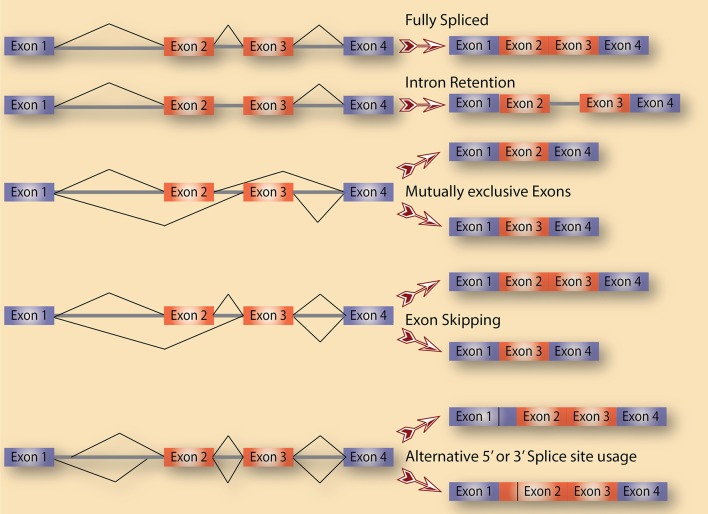
Alternative splicing as a source of diversification. A schematic diagram that depicts the various types of alternative splicing events that potentially exist in cells.

Alternative splicing is a term used to collectively refer to several splicing events. As shown in Figure 1, there are various distinct forms of alternative splicing, including alternative exons (cassette exons: skipped/included whole exons), retained introns, and alternative 5′ and 3′ splice sites (5′ ss and 3′ ss). There are also several less obvious alternative splicing events that are tightly coupled to and could be a consequence of transcription regulation such as alternative first and last exons. Nevertheless, all these events are well documented in eukaryotes with remarkable impacts on transcriptome diversification. One class of alternative splicing, intron retention, is often overlooked because it is interpreted as a splicing mistake that lead to an intron not being spliced out. While this might be true in several cases, a lot of evidence points to intron retention being regulated to control the expression of genes post-transcriptionally. In fact, cancer cells of all types are characterized by high levels of retained intron, leading to a higher diversity of their transcriptomes compared to normal cells (Dvinge and Bradley, 2015).

Intron retention and its regulation are obvious in a class of introns referred to as minor or U12 introns, which are conserved in almost all eukaryotes. Unlike the vast majority of introns in cells, which rely on the canonical spliceosome that is composed of U1, U2, U4, U5, and U6 snRNPs for their splicing, minor introns utilize a less abundant and seemingly less efficient spliceosome that is made of U11, U12, U4atac, U5, and U6atac snRNPs. Around 800 minor introns in the human genome are embedded in genes that function in signal transduction and information relay, cell cycle control and DNA damage repair (Turunen et al., 2013). We previously showed that hundreds of U12 introns are extremely conserved and are used as molecular switches that provide rapid control of gene expression that does not depend on transcription of new pre-mRNA especially when the gene product is needed instantaneously such as when cells are under stress (Younis et al., 2013). Given the functions of the genes that host minor introns, it is likely that they are regulated in a similar fashion in cancer.

## Mechanisms of splicing regulation

It is of note that some documented alternative splicing events constitute only a small fraction of the processed mRNA that are expressed at any given time. While this suggests that such alternative splicing events represent the expected biological noise of a process that is extremely active in cells, we argue that these events are tightly regulated and serve significant roles in various cell types and tissues. More specifically, the low abundance of these events in one cell type could have evolved to be so because the encoded protein from these specific splicing isoforms have a cell type- or condition-specific function. In addition, some of these events are only expressed at a high level when cells are faced with certain environmental conditions, such as stress, in when the specific splicing isoform becomes absolutely required (Younis et al., 2013). Thus, exhaustive searches are now needed to identify these conditions in which these isoforms become abundant and their function more significant. Finally, some disease tissues show enrichment of these events, suggesting specific functions. This infers that the abundance of specific alternatively spliced transcripts as well as the choice of the specific alternative splicing events for a given pre-mRNA are under tight regulation. This regulation is dictated by both cis-elements in the pre-mRNA itself and trans-factors such as RNA binding proteins (RBPs). The fact that the human genome encodes for thousands of RBPs, of which a big fraction functions in RNA splicing and its regulation strongly supports the notion that alternative splicing is not random but rather a highly regulated process and a key step in gene expression regulation.

Splicing factors historically have been classified into hnRNPs, which typically suppress splicing, and SR proteins, which tend to have a positive role in splicing regulation. However, a more thorough analysis of the function of any given hnRNP or SR protein quickly reveals that they do not always conform to these classifications. The ultimate role of an RBP in splicing regulation depends on multiple factors. These include the strength and context of its binding sites on the pre-mRNA in addition to either competitive or cooperative binding of multiple RBPs on or around the regulated exon or intron. This combinatorial regulation makes it very hard to predict the splicing outcome of reduced or increased binding of a single splicing factor in normal or diseased cells. Another complication is that a splicing factor is likely to regulated pre-mRNA splicing of other splicing factors in endless feedback loops and complex networks. A better approach to understand the regulation of alternative splicing in a given condition requires a systems biology approach in which the expression status and targets of multiple if not all RBPs be assessed to start building these networks of co-regulated pathways.

## Deregulation of alternative splicing in cancer

All data to date indicate that alternative splicing is a well-designed process that is tightly regulated in order to produce a network of alternatively spliced transcripts, which we refer to in this review as the splice-ome (spliceome). Work in the last two decades have moreover showed that the spliceome is significantly altered in disease states, such as cancer (Reviewed in, David and Manley, 2010; Chabot and Shkreta, 2016; Scotti and Swanson, 2016). In fact, every hallmark of cancer can be represented by several examples of proto-oncogenes, tumor suppressor genes, or other genes whose splicing is altered to produce isoforms that are needed for the transformation process (Oltean and Bates, 2014). In this review, we do not aim to provide a comprehensive list of all the cancer-related abnormal alternative splicing events, but rather highlight a few that exemplify a deregulated splicing program in cancer that is not a byproduct of the cancer phenotype but a driving force in cancer development and maintenance (Table 1). We thus discuss four main categories of splicing aberrations: (1) Cancer-specific splicing alterations in oncogenes and tumor suppressor genes. (2) Cancer-specific mutations in splicing factors. (3) Changes in upstream signaling pathways that deregulate splicing factors. And (4) aberrations in spliceosomal components that are linked to cancer. The involvement of alternative splicing in the 10 hallmarks of cancer has been reviewed elsewhere (Sveen et al., 2016). Here we summarize some of these changes to point to the fact that the cancer phenotype in several cancer types is heavily reliant on altering one or several splicing choices.

**Table 1:** Selected examples of genes with cancer-related alternatively spliced isoforms.

**Gene name protein product**	**Alterations in cancer-related functions due to alternative splicing**	**Cancer-related alternative splicing**	**References**
*TP53*/p53	Reduced anti-proliferative response to stress. Reduced tumor suppression	◦ Alternative 1st exon ◦ Intron 9 retention ◦ Intron 9 cryptic exon inclusion ◦ Intron 2 retention ◦ Cassette Exon 8 ◦ Intron 7 alternative 5′ ss	Surget et al., 2013
*Bcl2l1*/BclX	Apoptosis regulator: Anti-apoptotic (BCL-X_L_) Pro-apoptotic (BCL-X_S_)	◦ Intron 2 alternative 5′ ss	Paronetto et al., 2016
*CASP2*/Caspase 2	Apoptosis regulator: Anti-apoptotic (Casp2S) Pro-apoptotic (Casp2L)	◦ Cassette Exon 9	Jang et al., 2014
*AIMP2*/Aimp2	Apoptosis regulation: Anti-apoptotic (AIMP2-DX2) Pro-apoptotic (AIMP2-full length)	◦ Cassette Exon 2	Choi et al., 2011
*BIN1/* Myc box-dependent-interacting protein 1 (Bin1)	Reduced tumor suppression and apoptosis regulation	◦ Cassette Exon 12a ◦Cassette Exon 13	Anczuków et al., 2012
*TERT*/telomerase reverse transcriptase	Reduced replicative senescence Loss of telomerase activity in α,β, γ deletion isoforms	◦ Intron 5 alternative 3′ ss (α deletion) ◦ Cassette Exons 7-8 (β deletion) ◦ Cassette Exon 11 (γ deletion) ◦Combinations of α,β, and γ deletions	Liu et al., 2017
*CD44*/CD44 antigen	Imbalanced regulation of cell division, migration and adhesion	◦ Cassette Exon 18 ◦ Cassette Exon v6 ◦ Cassette Exons v8-10 ◦ Cassette Exons v4-5 ◦ Cassette Exons v4-7	Prochazka et al., 2014
*IRF3*/Interferon regulatory factor 3	Reduced cell growth inhibition Reduced cellular senescence through p53 activation	◦ Cassette Exon 2 ◦ Cassette Exon 3 ◦ Cassette Exon 6 ◦ Combination of Exons 2,3, and 6 skipping	Li et al., 2011
*RAC1*/Ras-related C3 botulinum toxin substrate 1	Reduced cell growth and cell cycle regulation Reduced cell-cell adhesion formation and contact inhibition	◦ Cassette Exon 3b	Singh et al., 2004; Radisky et al., 2005
*STAT3*/Signal transducer and activator of transcription 3	Dominant negative regulation of transcription (STAT3β)	◦ Intron 22 alternative 3′ ss	Caldenhoven et al., 1996; Zammarchi et al., 2011
*CDH11*/Cadherin11	Enhances invasion when the splice isoform (with unique intracellular domain) is expressed with WT Cadherin 11	◦ Intron 13 alternative (incomplete) splicing	Feltes et al., 2002
*FGFR2*/Fibroblast growth factor receptor 2	Promotes EMT and metastasis	◦ Mutually exclusive exon 8 (IIIb) or 9 (IIIc)	Wagner et al., 2003; Zhao et al., 2013; Abou-Fayçal et al., 2017
*KLF6*/Krüppel-like factor 6	Antagonism of tumor suppressor activity	◦ Intron 2 alternative 5′ ss ◦ Cassette Exon 3	DiFeo et al., 2009
*MST1R*/Macrophage-stimulating protein receptor or RON (Recepteur d'origine Nantais)	Increased cell motility and invasion	◦ Exon 11 skipping with Exon 5 and 6 Inclusion ◦ Exon 5 and 6 skipping with Exon 11 Inclusion ◦ Exon 5, 6, and 11 skipping ◦ Exon 6 skipping and Exon 5 inclusion ◦Partial Exon 5 and 6 splicing	Zhou et al., 2003; Eckerich et al., 2009; Chakedis et al., 2016
*VEGFA*/Vascular endothelial growth factor A	Enhanced pro-angiogenic function	◦ Cassette Exons 6 and 6b ◦ Cassette Exon 7 and 7b ◦ Alternative Intron 6 5′ ss ◦ Alternative Intron 6 3′ ss ◦ Cassette Exon 8	Pritchard-Jones et al., 2007; Harper and Bates, 2008
*CASP8/*Caspase-8	Reduced tumor suppression and pro-apoptotic activity	◦ Cassette Exon 8 ◦ Alternative Exon 8 and 9 splicing (136 bp insertion between exon 8 and 9)	Mohr et al., 2005; Olsson and Zhivotovsky, 2011
*FAS*/Tumor necrosis factor receptor superfamily member 6	Loss of Pro-apoptotic activity	◦ Cassette Exon 6 ◦ Cassette Exon 8 ◦ Intron 5 retention	van Doorn et al., 2002; Schwerk and Schulze-Osthoff, 2005; Tejedor et al., 2015
*MCL1*/Induced myeloid leukemia cell differentiation protein (Mcl-1)	Anti-apoptotic	◦ Cassette Exon 2	Shieh et al., 2009
*MDM2*/E3 ubiquitin-protein ligase (Mdm2)	Reduced p53 binding Enhanced tumor progression.	◦ Skipping of Exons 4-9 ◦ Skipping of Exons 4-11 ◦ Skipping of Exons 5-9 ◦ >40 isoforms	Bartel et al., 2002
*GLS*/Glutaminase	Deregulation of glutamate metabolism	◦ Intron 15 retention leading to cleavage and polyadenylation in Intron 15 and an isoform lacking the canonical last 4 exons.	van den Heuvel et al., 2012
*LDHC*/L-lactate dehydrogenase C chain	◦ Metabolic rescue in tumor cells ◦ Deregulation of cellular energetics	◦ Cassette Exon 3 ◦ Skipping of Exon 3-4 ◦ Skipping of exons 3, 6, and 7 ◦ Cassette Exon7	Koslowski et al., 2002
*MAX/* Protein max	Promotion of cell proliferation through enhanced glycolytic metabolism	◦ Cassette Exon 2 ◦ Cassette Exon 5	Babic et al., 2013
*PKM/* Pyruvate kinase PKM	◦ Tumor specific metabolism via ◦ PKM2 (fetal isoform with Exon10 inclusion)	◦ Mutually exclusive Exons 9 and 10	Chen et al., 2010; Zhang and Manley, 2013
*CCND1/* G1/S-specific cyclin-D1	Promotes cell proliferation	◦ Intron 4 retention leading to cleavage and polyadenylation in Intron 4 and an isoform lacking the canonical last exon	Paronetto et al., 2010
*EGFR/* Epidermal growth factor receptor	Oncogene	◦ ALternativer splicing of a combination of Exons9a, 10,16, or 17 ◦ Skipping of Exon 2-7 ◦ Skipping of Exons 2-22 ◦ …	Abou-Fayçal et al., 2017
*PTEN/* Phosphatidylinositol 3,4,5-trisphosphate 3-phosphatase and dual-specificity protein phosphatase (Pten)	Reduced tumor suppression	◦ Intron 3 retention ◦ Intron 5 retention ◦ Inclusion of partial Intron 5 ◦ Inclusion of partial Intron 3 ◦ Inclusion of 5′ end of intron H between Exons 8 and 9 ◦ Alternative splicing of the end of intron 5 to splicing acceptor site within intron E	Agrawal and Eng, 2006; Okumura et al., 2011

## Cancer-specific splicing alterations in oncogenes and tumor suppressor genes

Some of the earliest and most studied examples of alternative splicing events that lead to isoforms amiable for cancer are in genes involved in apoptosis such as the members of the Bcl2 family and several caspases. For example, intron 2 of the *Bcl2L1* gene, which encodes the Bcl-X protein, is alternatively spliced. More specifically, the spliceosome has a choice between two 5′ splice sites (5′ ss) for intron 2. Depending on which 5′ ss is chosen, the mRNA produced could be large (Bcl-XL), which encodes a Bcl-X protein with anti-apoptotic function, or small (Bcl-XS), encoding a Bcl-X protein that is missing an essential BH domain and is pro-apoptotic. Another example is Caspase 2 pre-mRNA splicing, whereas the spliceosome faces a choice of including exon 9, generating a caspase 2L mRNA or skipping exon 9, leading to the caspase 2S isoform. The large isoform encodes the pro-apoptotic Casp2L protein, whereas the anti-apoptotic Casp2S protein is encoded by the short isoform. Given that cancer cells are resistant to cell death by apoptosis, they need to ensure the production of Bcl-XS and/or Casp2L. In the absence of mutations in *Bcl2L1* and *Caspase 2* genes that would affect splice sites or other cis-elements leading to Bcl-XS and Casp2L production, cancer cells reprogram the splicing machinery and/or splicing factors that bind to these pre-mRNAs to ensure that the cancer-specific isoforms are enriched.

**Figure 2:**
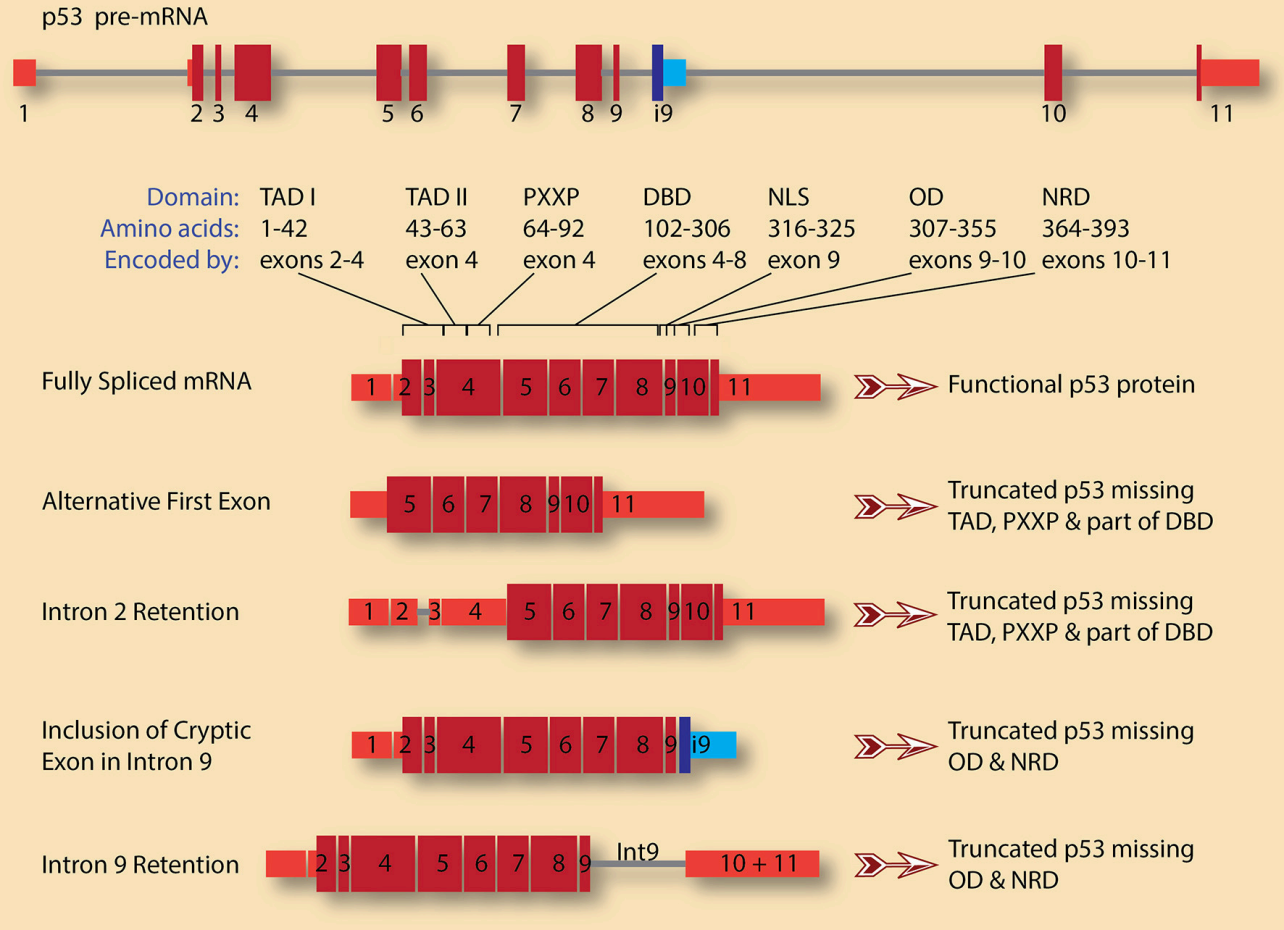
TP53 pre-mRNA splicing has significant consequences on the gene product, p53 protein. A schematic diagram of p53 pre-mRNA shows 11 exons (red boxes for canonical exons and blue boxes for cryptic intronic exon 9, i9) and 10 introns (gray lines). The size and shade of the exon indicate whether it is untranslated region (narrow, light red) or coding (wide, dark red). The various domains and the exons encoding them are also indicated (adopted from Surget et al., 2013). These are: Transactivation domains I and II (TAD I and TAD II), proline-rich region (PXXP), DNA-binding domain (DBD), nuclear localization signal (NLS), oligomerization domain (OD), and negative regulation domain (NRD). A fully spliced mRNA containing the 11 canonical exons encodes for full length and functional p53 protein. Several isoforms with various alternative first exons can be generated for p53 pre-mRNA. In this case, the first exon is what is usually exon 5 in the canonical transcript, leading to the production of p53 protein lacking TADs, PXXP, and part of DBD. This truncated p53 is expected to be dominant negative. A similar protein can be encoded by transcripts in which intron 2 is retained, leading to the usage of start codon in exon 4 rather than the canonical start codon in exon 2. On the other hand, retention of intron 9 and/or inclusion of the cryptic intronic exon 9, i9, change the reading frame causing the loss of the encoded amino acids from exons 10 and 11. The resulting p53 proteins lack OD and NRD. These truncated p53 proteins could compete with wild type p53 for DNA binding but are not functional as they cannot oligomerize.

Several tumor suppressor genes undergo alternative splicing in cancer that leads to either complete or partial loss of function. For example, complex alternative splicing of TP53, which encodes the p53 protein, generates several isoforms with significant impact on the protein function (Surget et al., 2013). Once activated, by DNA damage for example, p53 can induce cell-cycle arrest in either the G1 or G2 phase of the cell cycle. p53 can also activate Growth Arrest and DNA Damage 45 (GADD45), which regulates cell-cycle arrest in the G2/M phases. Thus, the presence of a functional p53 is essential for the multiple cell cycle checkpoints that allow cells to repair DNA damage or commit to apoptosis. Some of the protein products from the TP53 splicing isoforms are dominant negative, and since p53 acts as a tetramer, the production of these dominant negative subunits, even at low level, can have dramatic effects as they act as poison subunits. Four isoforms of these p53 transcripts are depicted in Figure 2.

Interestingly, even tumor viruses take advantage of alternative splicing to produce oncoproteins that cause host cell transformation. For example, the production of the two Human Papilloma Virus (HPV) oncoproteins E6 and E7 in patient tissues, which are encoded by one pre-mRNA, depends on alternative splicing. Briefly, unspliced transcripts (that is, the intron is retained) produce the E6 mRNA (and ORF) whereas complete splicing of the pre-mRNA produces the E7 mRNA and ORF. Other transcripts including E6^∧^E7 or E6^*^III are generated due to alternative 3′-splice usage (Graham and Faizo, 2017).

These few examples underscore the capacity of alternative splicing to produce two or more proteins from a single gene that could have completely opposite functions with major consequences on cell fate and the transformation process.

## Cancer-specific mutations or alterations in splicing factors

Given that cancer cells do reprogram the spliceome, it is not surprising that splicing factors are common targets for deregulation in this disease (Dvinge et al., 2016). These include ESRP1 and ESRP2 (Warzecha et al., 2009), hnRNP A1, hnRNP A2, hnRNP A2/B1, hnRNP H, hnRNP K, and hnRNP M (Moran-Jones et al., 2009; David et al., 2010; Golan-Gerstl et al., 2011; Lefave et al., 2011; Xu et al., 2014; Gallardo et al., 2015), PRPF6 (Adler et al., 2014), PTBP1(Izaguirre et al., 2012), QKI (Zong et al., 2014), RBFOX2 (Shapiro et al., 2011), RBM4, RBM5, RBM6, and RBM10 (Bonnal et al., 2008; Fushimi et al., 2008; Shapiro et al., 2011; Izaguirre et al., 2012; Bechara et al., 2013; Wang et al., 2014; Hernández et al., 2016), as well as SRSF1, SRSF2, SRSF3, SRSF6, and SRSF10 (Karni et al., 2007; Anczuków et al., 2012; Tang et al., 2013; Jensen et al., 2014; Zhou et al., 2014; Kim et al., 2015).

The SR protein SRSF2, for example, is a splicing factor that is commonly mutated in a collection of neoplastic diseases or cancers of immature blood cells known as Myelodisplastic Syndromes (MDS). Interestingly, mutations in SRSF2 that alter its sequence specificity on its target pre-mRNAs are more likely to be linked to MDS than nonsense mutations, indicating that a gain-of-function (binding to differential pre-mRNA targets) rather than loss-of-function of SRSF2 produces a new set of alternatively spliced mRNAs that are relevant to MDS development (Kim et al., 2015).

Of note, not all changes in splicing factors are due to mutations in their encoding genes as mutation-free disruptions in the repertoire of RNA-binding proteins (splicing factors) due to the imbalance in their expression is emerging as a common feature in many diseases including cancer. For example, frequent upregulation of mutation-free SRSF2 is a driver in the development of Hepatocellular Carcinoma (HCC) (Luo et al., 2017). SRSF1, also a splicing factor, is itself an oncogene whose expression is increased in cancers, including breast cancer (Das and Krainer, 2014; Akerman et al., 2015; Anczuków et al., 2015). These alterations in splicing factors, whether due to mutations or altered expression, tend to have large effects on cell phenotype as these splicing factors bind to and regulate the splicing of hundreds of pre-mRNAs. Thus, cancer cells can alter the splicing of a large number of genes by deregulating a handful of splicing factors. While this might seem to be an overkill, evidence does point to the fact that among the thousands of changes, some have distinct and significant effects on the transformation process. For example, SRSF2 mutants in MDS lead to mis-splicing of hundreds of pre-mRNAs, but one of them, the EZH2 pre-mRNA, encoding a transcriptional regulator that is required for maintaining the repressed state of many genes during hematopoiesis, stands out. Hematopoietic cells expressing SRSF2 mutants show higher inclusion of a highly conserved “poison” exon in the EZH2 mRNA, leading to degradation of the mRNA by nonsense-mediated decay and loss-of-function of the *EZH2* gene (Kim et al., 2015).

hnRNP proteins also play their share in cancer progression. For example, mis-regulation of a number of hnRNP proteins have been linked to HCC tumor progression, whereas the overexpression of hnRNP A1 in particular has been linked to tumor invasion and metastasis (Zhou Z. J. et al., 2013). The detailed contribution of the RNA splicing-dependent effects of mis-regulation of many hnRNPs in cancer is still under intense investigation by several laboratories and should shed some light on mechanisms as well as potential novel therapeutic targets.

Of note here is that mutations in splicing factors in MDS patients typically cause distinct and sometimes non-overlapping splicing defects, suggesting an alternate underlying mechanism. Indeed, a recent study has uncovered that mutations on distinct splicing factors in MDS commonly cause elevated R-loops, replication stress, and activation of the ataxia telangiectasia and Rad3-related protein (ATR)-Chk1 pathway (Chen et al., 2018). These effects can lead to deregulated transcription pause release, raising the possibility that the MDS phenotype is related to a transcriptional defect rather than a splicing one.

## Changes in upstream signaling pathways that deregulate splicing factors

In order to ensure that several splicing factors and other cellular processes are deregulated, the signaling pathways that relay extracellular signals to splicing factors are often targeted in cancer. The SR protein family is often deregulated as the function of SR proteins tightly depends on their phosphorylation status, which itself is regulated by upstream kinases. For example, the splicing of the cassette exons in Caspase 9 pre-mRNA is regulated by the splicing factor SRSF1 leading to either caspase 9a or caspase 9b mRNAs. SRSF1 is itself phosphorylated upon activation of multiple signaling pathways, including the PI3K/AKT pathway. Since AKT signaling is often constitutively activated in cancers, such as lung cancer, this leads to constitutive phosphorylation of SRSF1 and deregulated expression of Caspase 9a/9b (Shultz et al., 2010). A similar pathway involving an AKT-hnRNP U axis has also been shown to regulate Caspase 9a/9b ratio (Vu et al., 2013). This deregulated Caspase 9a/9b ratio has marked consequences on apoptosis and contributes to the ability of cancer cells to resist cell death.

Interestingly, several key components of signaling pathways that are typically deregulated in cancer can themselves be alternatively spliced to produce cancer-specific isoforms. For example, the inclusion of exon 6 in the pre-mRNA of the First Apoptosis Signal (Fas) receptor produces an isoform that encodes a membrane bound receptor that plays a key role in relaying extracellular signal that lead to programmed cell death. On the other hand, the Fas isoform with exon 6 being skipped encodes a soluble protein that does not induce apoptosis upon relevant signaling. Epidermal Growth Factor Receptor (EGFR), Insulin Receptor (INSR), Receptor d'Origine Nantais (RON), and Vascular Endothelial Growth Factor Receptor (VEGFR) are among several receptor tyrosine kinases whose splicing is altered in cancer leading to tumor progression or reduced response to therapy (reviewed in, Abou-Fayçal et al., 2017). In the case of VEGFR, one intron retention leads to the production of a shorter and decoy receptor that is dominant negative (Kendall et al., 1996; Vorlová et al., 2011). Similarly, alternative splicing in EGFR pre-mRNA produces several isoforms, some of which are dominant negative whereas others are constitutively active, leading to enhanced tumorgenicity, migration and invasion (Guillaudeau et al., 2012a,b; Piccione et al., 2012; Zhou M. et al., 2013; Zhou Z. J. et al., 2013; Padfield et al., 2015).

## Aberrations in spliceosomal components that are linked to cancer

It is remarkable that loss-of-function mutations in core components of the spliceosome are not compatible with life, which speaks to the critical role the spliceosome plays in all cells. However, components of the spliceosome can be mutated without complete loss-of-function leading to widespread alterations in splicing and disease.

Patients with MDS, chronic myelomonocytoic leukemia (CMML), or chronic lymphocytic leukemia (CLL) acquire mutations in the spliceosomal components SF3B1, SF1, PRPF40B, and U2AF35 besides mutations in the splicing factor SRSF2 and ZRSR2, a component of U11/U12 di-snRNP, (Armstrong et al., 2018). Interestingly, SF3B1 and U2AF35 mutation tend to be missense mutations and mutually exclusive, again suggesting that cells with severe aberrations in spliceosome function are not viable (Armstrong et al., 2018). These mutations are drivers in cancer and they strongly correlate with prognosis and clinical phenotype.

On the other hand, several genetic diseases are linked to mutations in core components of the spliceosome. These include retinitis pigmentosa, a progressive neurodegeneration of Rod photoreceptors in the retina, which is linked to mutations in PRPF31, PRPF8, BRR2, PRPF4, or PRPF3. Spinal Muscular Atrophy (SMA) is a severe neurodegenerative disease caused by mutations in *SMN1* gene, which encodes a protein that functions in the biogenesis of spliceosomal snRNPs and reduced SMN function in cells has been shown to lead to widespread aberrations in splicing. Mutations in one of the snRNA components of the minor spliceosome, U4atac, have been identified and linked to severe mental retardation and dwarfism, microcephalic osteodysplastic primordial dwarfism type 1 (TALS/MOPD). Despite their low abundance in cells, minor introns are highly conserved and serve as critical molecular switches for the expression of genes that harbor them (Younis et al., 2013). Some of these genes are bona fide oncogenes and tumor suppressor genes, suggesting a role for deregulating minor intron splicing in cancer (Unpublished data).

## Systematic approaches for identifying splicing aberrations that are linked to cancer

Genetic alterations or mutations in cancer patients that affect the splicing of one gene are relatively easy to study, track, and even propose therapeutic tactics based on fixing the splicing of that one pre-mRNA. However, a major challenge emerges when the alteration is in a splicing factor or core component of the spliceosome as these lead to global (thousands) alterations in splicing affecting a wide range of genes. Still more challenging are cases where the expression of the splicing factors is altered without an obvious underlying genetic mutation. Two of the many challenges are: (1) identifying amongst the thousands of splicing alterations those that significantly contribute to cancer progression, and (2) therapeutically target the splicing factors without having massive side effects that sometimes are worse than the actual disease itself. In order to successfully address these points, it is important to develop a systematic and standardized approach to gain sufficient understanding of splicing deregulation in cancer and their impact on cancer.

Before the advent of next generation sequencing of RNA (referred to here as RNA-seq), transcriptome profiling to identify global splicing changes relied mostly on Gene Expression Exon Microarrays, that contain probes for almost all exons and many introns. While these arrays were a major advance over traditional microarrays with limited number of probes per gene, they tend to be very hard to interpret and generate a lot of false positives if not properly analyzed and replicated. Also, the lack of a large number of probes in introns causes these microarrays to miss a major category of splicing aberration that is intron retention. Nowadays, the gold standard for transcriptome profiling that includes both information on genome-wide expression level changes as well as splicing disruptions is RNA-seq. While this method is both quantitative and qualitative, it has its own challenges as well. As a start, generating the libraries for RNA-seq from high quality RNA is expensive, tedious, and require well trained personnel. The data generated is massive in size (several gigabytes per sample) and requires powerful computing machines for storage as well as analysis. The analysis itself is a major bottleneck. Several off-the-shelf pipelines exist for mapping raw reads and analysis of differential expression of genes. However, these only scratch the surface and do not fully take advantage of the wealth of data generated by any well-designed RNA-seq experiment. For example, there are several publicly available algorithms that attempt to identify splicing alterations from RNA-seq, but our personal experience with these is that they all fail at capturing the real picture as most of them use statistical models that are not suitable for biological systems, leading to identification of an endless list of statistically significant but small changes that have no or little impact on the phenotype. Thus, many laboratories have opted for their own in-house pipelines that are suited for their own analysis but remain far from suited to apply globally. The major challenge in the analysis of RNA-seq data does not detract from the fact that it has been widely used to generate important databases of splicing alterations in many cancers. The list of splicing aberrations in cancer will grow and our understanding of the molecular basis of these changes as well as their contribution to cancer will improve tremendously in the coming years as our ability to standardize the analysis pipeline improves. In fact, we propose that identifying the right splicing isoforms can be so powerful, they should be used as novel biomarkers for many cancer types and subtypes.

Once enough molecular understanding of the splicing aberrations is gained and their impact on cancer is proven, innovative RNA-based therapies are required to correct the splicing alterations or induce splicing changes in cancer cells that make them more susceptible to traditional chemotherapy. Only recently, RNA-based therapies, which include a range of mechanisms such as antisense oligonucleotides, RNAi, anti-miRNA, miRNA mimics, aptamers, ribozymes, and others, seemed far-fetched and unpractical. However, the recent success of antisense oligonucleotides in correcting the splicing of exon 7 of *SMN* gene in SMA patients and its approval by the FDA speaks to the power of such therapies. In order to start applying such strategies to cancer, it is better to focus first on a few targets with large effects on phenotype. For example, given the large contribution of mis-splicing of genes involved in apoptosis on the ability of cancer cells to resist cell death, these genes are the low hanging fruit. In addition, it is noted that splicing alterations would rarely be the sole driver in cancer progression, we thus do not suggest the use of RNA-based therapies to overcome splicing aberrations as an alternate to traditional therapies, but rather a combination therapy for more effective treatment (we anticipate the effects to be synergistic) with less unfavorable side effects.

## Author contributions

EE curated and compiled information toward completion of this review. IY wrote the review.

### Conflict of interest statement

The authors declare that the research was conducted in the absence of any commercial or financial relationships that could be construed as a potential conflict of interest.
